# Effect of Roux‐en‐Y gastric bypass on liver mitochondrial dynamics in a rat model of obesity

**DOI:** 10.14814/phy2.13600

**Published:** 2018-02-21

**Authors:** Jessica Sacks, Anny Mulya, Ciaran E. Fealy, Hazel Huang, John D. Mosinski, Mangesh R. Pagadala, Hideharu Shimizu, Esam Batayyah, Philip R. Schauer, Stacy A. Brethauer, John P. Kirwan

**Affiliations:** ^1^ Department of Pathobiology Lerner Research Institute Cleveland Clinic Cleveland Ohio; ^2^ Molecular Medicine Cleveland Clinic Lerner College of Medicine Case Western Reserve University Cleveland Ohio; ^3^ Department of Gastroenterology and Hepatology Digestive Disease Institute Cleveland Clinic Cleveland Ohio; ^4^ Bariatric and Metabolic Institute Cleveland Clinic Cleveland Ohio; ^5^ Metabolic Translational Research Center Endocrinology and Metabolism Institute Cleveland Clinic Cleveland Ohio

**Keywords:** Biogenesis, Fusion and Fission, Hepatic steatosis, Mitochondria, Mitophagy

## Abstract

Bariatric surgery provides significant and durable improvements in glycemic control and hepatic steatosis, but the underlying mechanisms that drive improvements in these metabolic parameters remain to be fully elucidated. Recently, alterations in mitochondrial morphology have shown a direct link to nutrient adaptations in obesity. Here, we evaluate the effects of Roux‐en‐Y gastric bypass (RYGB) surgery on markers of liver mitochondrial dynamics in a diet‐induced obesity Sprague‐Dawley (SD) rat model. Livers were harvested from adult male SD rats 90‐days after either Sham or RYGB surgery and continuous high‐fat feeding. We assessed expression of mitochondrial proteins involved in fusion, fission, mitochondrial autophagy (mitophagy) and biogenesis, as well as differences in citrate synthase activity and markers of oxidative stress. Gene expression for mitochondrial fusion genes, mitofusin 1 (*Mfn1*;* P* < 0.05), mitofusin 2 (*Mfn2*;* P* < 0.01), and optic atrophy 1 (*OPA1*;* P* < 0.05) increased following RYGB surgery. Biogenesis regulators, peroxisome proliferator‐activated receptor gamma coactivator 1‐alpha (*PGC1α*;* P* < 0.01) and nuclear respiratory factor 1 (*Nrf1*;* P* < 0.05), also increased in the RYGB group, as well as mitophagy marker, BCL‐2 interacting protein 3 (*Bnip3*;* P* < 0.01). Protein expression for Mfn1 (*P* < 0.001), PGC1*α* (*P* < 0.05), BNIP3 (*P* < 0.0001), and mitochondrial complexes I‐V (*P* < 0.01) was also increased by RYGB, and Mfn1 expression negatively correlated with body weight, insulin resistance, and fasting plasma insulin. In the RYGB group, citrate synthase activity was increased (*P* < 0.02) and reactive oxygen species (ROS) was decreased compared to the Sham control group (*P* < 0.05), although total antioxidant capacity was unchanged between groups. These data are the first to show an association between RYGB surgery and improved markers of liver mitochondrial dynamics. These observed improvements may be related to weight loss and reduced energetic demand on the liver, which could facilitate normalization of glucose homeostasis and protect against hepatic steatosis.

## Introduction

The chronic imbalance in nutrient supply and demand is a hallmark characteristic of obesity and type 2 diabetes (T2D) (Liesa and Shirihai 2013). Bariatric surgery is considerably more effective in restoring balance compared to medical therapy, and this is evidenced by several long‐term prospective and randomized controlled trials showing sustained weight loss and glycemic control in obese patients (Mingrone et al. 2012, 2015; Schauer et al. 2012, 2017; Kashyap et al. 2013). In addition to striking improvements in body weight and insulin sensitivity, Roux‐en‐Y gastric bypass (RYGB) has also been shown to reverse non‐alcoholic fatty liver disease (NAFLD) and related steatosis, inflammation, and fibrosis (Sasaki et al. 2014; Bower et al. 2015). We have previously shown that bariatric surgery reduces insulin resistance and hepatic steatosis and inflammation from obese, non‐diabetic Sprague‐Dawley (SD) rats, and protects against endoplasmic reticulum (ER) stress and apoptosis (Mosinski et al. 2016). The specific cellular and molecular mechanisms by which re‐routing of the gut anatomy facilitates metabolic improvements in the liver is an area of active investigation.

Recent reports suggest a direct link between ER stress and mitochondrial dynamics (Malhotra and Kaufman 2011; Marchi et al. 2014). The latter is defined as the continual fusion and division of mitochondria as part of their normal quality control mechanism. Mitochondrial fusion is mediated by the mitofusin proteins Mfn1 and Mfn2, and optic atrophy 1 (OPA1), while fission is primarily regulated by dynamin‐related protein 1 (Drp1), fission protein 1 (Fis 1), and mitochondrial fission factor (MFF). When cells are over‐exposed to excess nutrients, as is common in obesity, mitochondria accumulate damage and tend to become more fragmented via increased fission (Liesa and Shirihai 2013; Wai and Langer 2016). Damaged mitochondria are tagged for mitochondrial autophagy (mitophagy) by proteins, Parkin and PTEN‐induced putative kinase (PINK1) (Shirihai et al. 2015). BCL‐2 interacting protein 3 (BNIP3) is another regulator of mitophagy in the liver, and has recently been shown to prevent steatohepatitis (Glick et al. 2012). In contrast, mitochondria remain more elongated in cells under nutrient deprivation, via mechanisms of increased fusion (Wai and Langer 2016). Disruptions in fusion‐dependent mechanisms have been shown to occur in obesity, including reduced skeletal muscle Mfn2 expression in obese Zucker rats and humans compared to lean controls (Bach et al. 2003). Furthermore, liver‐specific ablation of Mfn2 in mice has been shown to markedly increase hepatic ER stress, and result in several metabolic abnormalities, including impaired glucose tolerance and insulin resistance (Sebastian et al. 2012). However, it is unknown whether bariatric surgery modulates liver mitochondrial dynamics, and whether this is further modified in the setting of high‐fat diet‐induced obesity. In addition to fusion, fission, and mitophagy, mitochondrial biogenesis plays an essential role in regulating mitochondrial quality control. The transcription factor, peroxisome proliferator‐activated receptor gamma coactivator 1‐alpha (PGC1*α*), and citrate synthase activity are two common markers for mitochondrial biogenesis, and have each been shown to increase in skeletal muscle (Gastaldi et al. 2007) and adipose tissue (Jahansouz et al. 2015) following bariatric surgery. Two downstream transcription factors of PGC1*α*, nuclear respiratory factor 1 (NRF1) and mitochondrial transcription factor A (Tfam), are also critical components in driving mitochondrial DNA replication (Jornayvaz and Shulman 2010). Whether RYGB surgery alters liver mitochondrial biogenesis and content in the presence of chronic high‐fat feeding has yet to be determined.

We investigated the effects of RYGB surgery on markers of mitochondrial dynamics in the liver of obese SD rats, and hypothesized that RYGB would increase expression of mitochondrial fusion proteins, decrease fission proteins and improve overall mitochondrial quality control mechanisms, consistent with improvements in glycemic control.

## Materials and Methods

### Animal care and surgical intervention

The protocol was approved and performed in compliance with the Cleveland Clinic Institutional Animal Care and Use Committee (IACUC), as noted previously (Mocanu et al. 2015; Mosinski et al. 2016). Briefly, sixteen 12‐week‐old SD rats (Charles River Laboratories, Wilmington, MA) were housed individually in a 12‐h light/dark cycle under constant ambient temperature (21–23°C) and humidity. Animals were provided an ad libitum high‐fat diet (D12492, 60% fat, Research Diets, New Brunswick, NJ) to establish diet‐induced obesity and insulin resistance. At 24–25 weeks of age, animals were randomized into Sham surgery (*N* = 8) or RYGB (*N* = 8). Animals were fasted overnight prior to surgery, and the gastric bypass procedure was performed as previously described (Gatmaitan et al. 2010). For the sham operation, the stomach and distal esophagus were exposed and dissected free for the same duration required for gastric bypass surgery. Rats recovered on an ad libitum liquid diet with Boost (Nestle, Buffalo Grove, IL) for up to 7‐days after surgery, and returned to an ad libitum HFD thereafter. All rats were euthanized 90 days postoperatively following a 16‐h overnight fast, and liver tissues were collected, snap frozen in liquid nitrogen, and stored at −80°C.

### RNA extraction

Liver RNA was extracted using the RNeasy Mini kit (Qiagen, Valencia, CA) by homogenizing 20 mg of liver tissue in buffer RLT with the FastPrep‐24 tissue homogenizer (MP Biomedicals, Santa Ana, CA). Isolated RNA was eluted in 40 *μ*L nuclease‐free water. Total RNA concentration and purity was measured by absorbance at 230, 260 and 280 nm on a NanoDrop ND‐1000 Spectrophotometer (Thermo Fisher Scientific). Purified RNA was aliquoted and stored at −80°C until further analysis.

### cDNA synthesis

Complementary DNA (cDNA) was generated by reverse transcription from 1 *μ*g of total RNA using the iScript cDNA synthesis kit (BioRad) and a PX2 Thermal Cycler (Thermo Fisher Scientific). Reaction volume was set at 20 *μ*L, and cDNA synthesis was performed at 25°C for 5 min, 42°C for 30 min, and 85°C for 5 min.

### qRT‐PCR Primer Pairs

Specific target gene primer pairs were obtained from PrimerBank database for rodents (Table 1). All primers were checked for specificity to genes of interest by performing a Blast analysis against the rat mRNA sequence.

**Table 1:** Gene‐specific primers for qRT‐PCR analysis

Gene	Forward	Reverse	GenBank Accession No.
MFN1	CCTTGTACATCGATTCCTGGGTTC	CCTGGGCTGCATTATCTGGTG	NM_138976.1
MFN2	GATGTCACCACGGAGCTGGA	AGAGACGCTCACTCACTTTG	NM_130894.4
OPA1	CAGCTGGCAGAAGATCTCAAG	CATGAGCAGGATTTTGACACC	NM_133585.3
DNM1L	AGGTTGCCCGTGACAAATGA	ATCAGCAAAGTCGGGGTGTT	NM_053655.3
FIS1	ACCACCGCCTTCCTTTTCTC	AAGCCACGGCCCAACTTTAT	NM_001105919.1
PINK1	CTGTCAGGAGATCCAGGCAATT	GCATGGTGGCTTCATACACAGC	NM_001106694.1
BNIP3	ACTTTGCAGTCCCCCTCTTC	AAAGGCGTAACACAACTGCC	NM_053420.3
TFAM	TCATGACGAGTTCTGCCGTT	AGAACTTCACAAACCCGCAC	NM_031326.1
NRF1	TACAAGGCGGGGGACAGATA	ACTCCATCTGGGCCATTAGC	NM_001100708.1
PPARGC1a	TATGGAGTGACATAGAGTGTGCT	CCACTTCAATCCACCCAGAAAG	NM_031347.1
ACTb	CGGTCAGGTCATCACTATCG	TTCCATACCCAGGAAGGAAG	NM_031144.3

Primer pairs for genes regulating mitochondrial dynamics and quality control. MFN1, Mitofusin 1; MFN2, Mitofusin 2; OPA1, Optic atrophy 1; DMN1L, Dynamin 1‐like; FIS1, Fission 1; PINK1, PTEN‐induced putative kinase 1; BNIP3, BCL2 interacting protein 3; TFAM, Transcription factor A mitochondrial; NRF1, Nuclear respiratory factor 1; PPARGC1a, PPAR‐gamma coactivator 1‐alpha; ACTb; Beta‐Actin.

### Semi‐quantitative RT‐PCR analysis

Determination of relative mRNA expression was conducted in duplicate on a QuantStudio 5 Real‐Time PCR system (Thermo Fisher Scientific) using 10 ng of template cDNA and VeriQuest SYBR Green qPCR Master Mix (Affymetrix, Santa Clara, CA). The rat *β*‐Actin gene was used as an internal standard for normalization, and relative changes in mRNA abundance were calculated using the comparative ΔΔCt method (Livak and Schmittgen 2001). Briefly, the threshold cycle (Ct) for *β*‐Actin was subtracted from the Ct for the gene of interest to determine the ΔCt value. Fold‐induction of target genes in RYGB animals was calculated by subtraction of RYGB ΔCt from the average Sham ΔCt, and expressed as an exponential of the negative value.

### Protein extraction and western blot analysis

Liver homogenates were prepared by grinding 50 mg of tissue in ice‐cold lysis buffer (Life Technologies, Carlsbad, CA) in the presence of protease inhibitor cocktail (Sigma Aldrich, St. Louis, MO), phenylmethylsulfonyl fluoride (Sigma Aldrich), sodium orthovanadate (Sigma Aldrich), and Phos‐STOP reagent (Roche Applied Sciences, Indianapolis, IN). Protein homogenates were centrifuged at 4°C for 10 min at 9,600 × *g*, supernatant was transferred to a new microcentrifuge tube, and lysates were stored at −80°C until further use. Protein concentration was measured in duplicate with a Protein BCA assay kit (Pierce Biotechnologies, Rockford, IL) and 25 *μ*g of total protein was prepared in Laemmli sample buffer containing 5% *β*‐mercaptoethanol and heated at 95°C for 5 min. Samples were separated by gel electrophoresis using a 4–20% Novex Wedgewell TrisGlycine SDS PAGE System (Life Technologies), and then transferred onto polyvinylidene fluoride membranes (Biorad, Hercules, CA). Membranes were blocked in 5% bovine serum albumin (BSA) in phosphate‐buffered saline containing 0.1% Tween 20 (PBST) for 30 min at room temperature. Membranes were incubated overnight at 4°C in primary antibody diluted 1:1000 (unless noted otherwise) in PBST for recognition of specific target proteins Mfn1 (R&D Systems, Minneapolis, MN), Mfn2 (Abnova, Jhongli, Taiwan), OPA1 (Abnova), Drp1 (Cell Signaling Technology, Danvers, MA), Fis1 (Thermo Fisher Scientific, Waltham, MA), PINK1 (Abcam, Cambridge, MA), Parkin (Cell Signaling Technology), BNIP3 (Cell Signaling Technology), total OXPHOS rodent cocktail (Abcam), or PGC1*α* (Santa Cruz Biotechnology, Dallas, TX), with HSC70 (1:5000; Santa Cruz Biotechnology) or Actin (1:5000; EnCor Biotechnology Inc., Gainesville, FL) as loading control. Membranes were washed with PBST and incubated in 1:5000 dilutions of anti‐rabbit, anti‐mouse (GE Healthcare, Piscataway, NJ) or anti‐sheep (Santa Cruz Biotechnology) secondary horseradish peroxidase‐conjugated antibodies for 1 h at room temperature. Immunoreactive proteins were detected by enhanced chemiluminescence reagent (Amersham ECL Prime; GE Healthcare) and quantified by densitometry using ImageJ software.

### Citrate synthase activity assay

Determination of liver citrate synthase activity was performed using the Citrate Synthase Assay Kit (Sigma Aldrich), which measures the formation of citric acid from acetyl coenzyme A (acetyl CoA) and oxaloacetic acid (OAA) via endogenous enzyme activity. Briefly, protein was extracted from 50 mg of liver tissue using a chilled glass homogenizer in 1 mL of CelLytic MT lysis reagent containing protease inhibitor cocktail and centrifuged for 10 min at 15,000*g* and 4°C. Clarified supernatants were aliquoted and stored at −80°C for later analysis. Samples were diluted 1:100 in 1x Assay Buffer containing 30 mmol/L Acetyl CoA and 10 mmol/L 5,5′‐Dithiobis‐(2‐nitrobenzoic acid) (DTNB) Solutions in duplicate. Baseline citrate synthase activity was measured spectrophotometrically on a SpectraMax 190 Microplate Reader (Molecular Devices, Sunnyvale, CA) at 412 nm on a kinetic program of 10 sec intervals for 1.5 min, before initiating the reaction by adding 10 mmol/L Oxaloacetate (OAA) Solution to each well. The absorbance of the reaction mixture was followed again using the same program, and total citrate synthase activity was measured by plotting the absorbance (A_412_) values against time for each reaction, and then calculating the change in absorbance in the linear range.

### In vitro reactive oxygen species (ROS) assay

Quantification of liver‐specific ROS was performed using the OxiSelect In Vitro ROS/RNS Assay Kit (Cell Biolabs Inc., San Diego, CA) according to the manufacturer's protocol. Briefly, rat liver tissue (50 mg) was homogenized in the same manner as for protein extraction, and purified supernatant was isolated and diluted 10‐fold in PBS. Diluted samples (50 *μ*L) were incubated for 5 min in duplicate with 50 *μ*L of catalyst reagent, and then with 100 *μ*L of the specific ROS probe, dichloro‐dihydrofluorescein DiOxyQ (DCFH‐DiOxyQ) solution for an additional 30 min in the dark at room temperature. The generated fluorescent product dichlorofluorescein (DCF) was measured on a SpectraMax M2 spectrophotometer (Molecular Devices) at a 480/530 nm excitation/emission wavelength, and ROS concentration was determined fluorometrically against hydrogen peroxide (H_2_O_2_) standards.

### Total antioxidant capacity assay

Evaluation of liver antioxidant capacity was conducted using the Total Antioxidant Capacity Assay Kit (Abcam), which utilizes the conversion of Cu^2+^ ions to Cu^+^ through endogenous protein and small molecule antioxidants, standardized to Trolox equivalents. Approximately 30 mg of liver tissue was homogenized in 800 *μ*L of ice‐cold PBS, and clarified protein supernatants were aliquoted following centrifugation as described above. Samples were diluted 1:50 in sterile deionized water, and 100 *μ*L of Trolox standard and protein samples were added to each well of a 96‐well plate. The reaction was initiated by adding 100 *μ*L of Cu^2+^ working solution to each well, and mixing the plate on an orbital shaker for 90 min at room temperature shielded from light. Colorimetric activity was measured by optimal density at 570 nm on a SpectraMax 190 Microplate Reader (Molecular Devices), and antioxidant capacity was calculated against the linear Trolox standard calibration curve.

### Statistics

All data are reported as mean ± standard deviation (STDEV). Statistical differences in gene and protein expression, citrate synthase activity, and ROS between the Sham and RYGB groups were analyzed using *t*‐test statistics. Correlations between protein expression and body weight, fasting plasma insulin, or HOMA‐IR were determined using linear regression models fit and the Pearson correlation coefficient. Differences between groups were considered significant at *P* < 0.05 and statistical tests were performed using GraphPad Prism 7.

## Results

### Gene expression in liver

**Figure 1:**
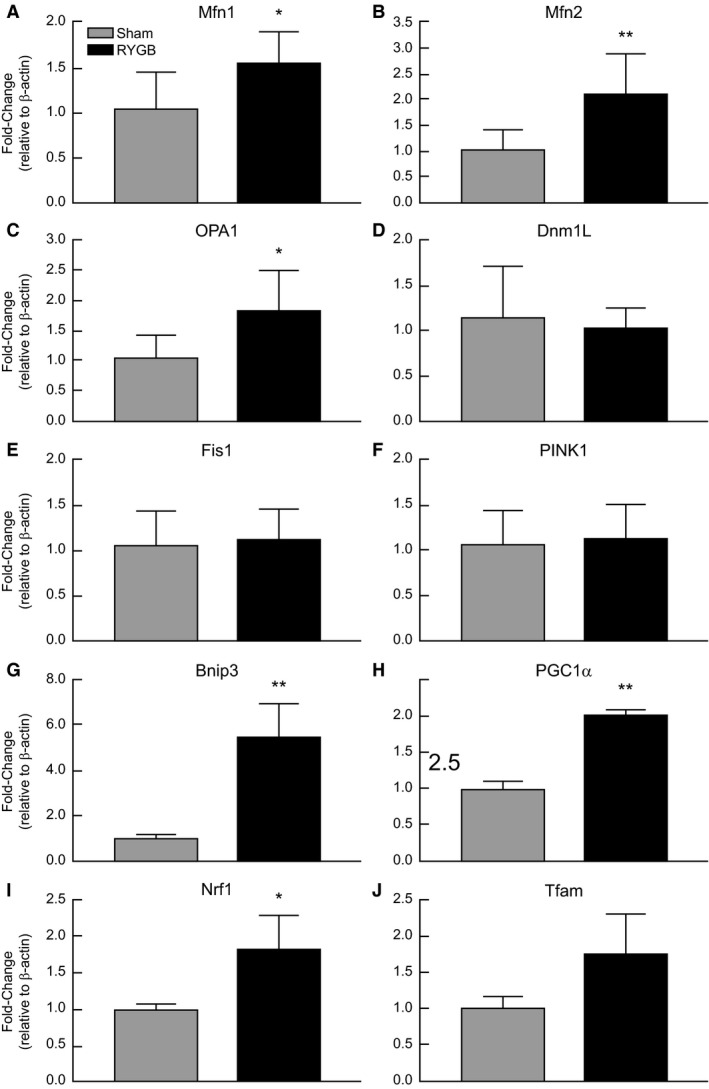
RYGB surgery results in increased mRNA expression of genes involved in mitochondrial fusion, biogenesis, and mitophagy in the liver of HFD‐induced obese rats. Fold induction of *Mfn1* (A), *Mfn2* (B), *OPA1* (C), *Dnm1L* (D), *Fis1* (E), *PINK1* (F), *Bnip3* (G), *PGC1α* (H), *NRF1* (I), *Tfam* (J) genes in liver tissue from Sham (N = 8) and RYGB (*N* = 8) animals. Data are expressed as mean ± STDEV. *P < 0.05 versus Sham control rats, **P < 0.01.

There was a significant increase in mRNA expression for fusion genes, *Mfn1* (*P* = 0.02), *Mfn2* (*P* = 0.003), and *OPA1* (*P* = 0.01) in livers of RYGB animals compared to Sham (Fig. 1A–C). While no between‐group differences were observed in either of the fission genes, dynamin‐1‐like (*DnmL1*; gene encoding for Drp1) and *Fis1* (Fig. 1 D–E), or the mitophagy gene, *PINK1* (Fig. 1F), there was a significant fivefold increase in Bnip3 (*P* = 0.007, Fig. 1G), as well as an induction in biogenesis markers, *PGC1α* (*P* = 0.007) and *NRF1* (*P* = 0.04) in the RYGB group versus Sham (Fig. 1H–I). Mitochondrial DNA transcription factor *Tfam* (*P* = 0.09) also trended toward increased mRNA expression in RYGB liver (Fig. 1J).

### Protein expression in liver

**Figure 2:**
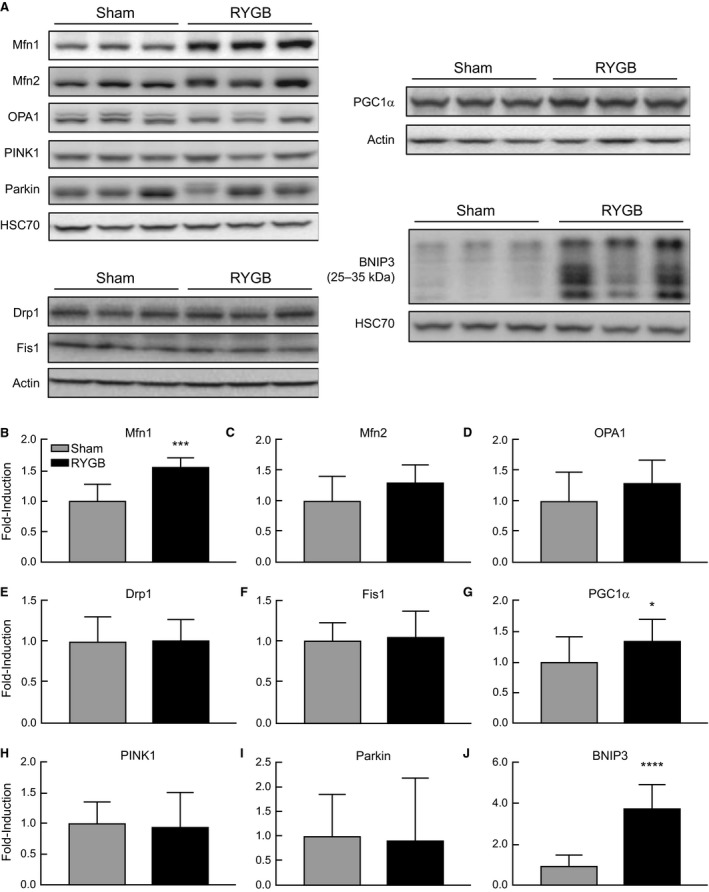
RYGB surgery results in increased expression of Mfn1, PGC1*α*, and BNIP3 in the liver of HFD‐induced obese rats. Representative western blot analysis (A) and quantification (B–J) of mitochondrial dynamics and quality control protein expression in Sham (*N* = 8) and RYGB (*N* = 8) animals. Data are expressed as mean ± STDEV. Fold‐inductions relative to HSC70 or Actin loading controls. *P < 0.05 versus Sham control rats, ***P < 0.001, ****P < 0.0001.

In addition to gene expression, we also assessed differences in mitochondrial dynamics protein expression at the translational level. There was a significant increase in Mfn1 (*P* = 0.0005) protein in liver lysates from RYGB animals compared to Sham (Fig. 2A–B). Fusion proteins, Mfn2 (*P* = 0.06) and OPA1 (*P* = 0.07) trended toward increased expression in the RYGB group as well. There were no differences in expression of mitochondrial fission proteins. Consistent with mRNA expression, we also observed a significant increase in PGC1*α* (*P* = 0.013) protein levels in the RYGB group versus Sham animals (Fig. 2A, G). Although there were no changes in expression of mitophagy markers, PINK1 or Parkin, we observed a significant induction in BNIP3 (*P* < 0.0001) expression in RYGB animals (Fig. 2A, J), similar to those seen at the transcriptional level.

### Citrate synthase activity and protein expression of mitochondrial complexes

**Figure 3:**
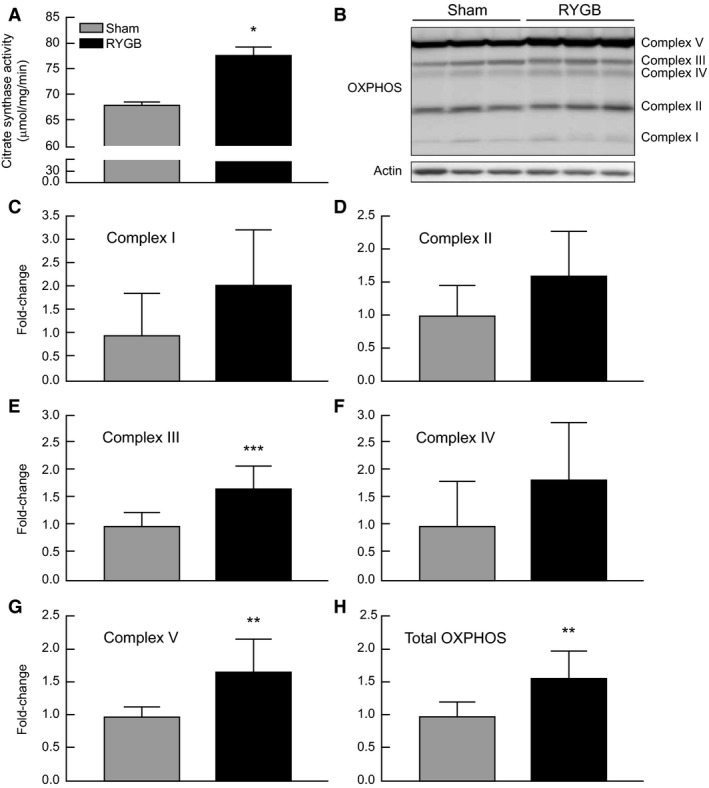
The effect of RYGB on markers of mitochondrial content in liver tissue from Sham (*N* = 8) and RYGB (*N* = 8) animals. Citrate synthase activity (A), Representative western blot image (B), and quantification (C–H) of respiratory chain complex protein expression. Data are expressed as mean ± STDEV. *P < 0.05 versus Sham control rats, **P < 0.01, ***P < 0.001.

Liver citrate synthase activity was significantly increased (*P* = 0.016) in RYGB animals versus Sham (Fig. 3A). Measurement of the five OXPHOS complexes by rodent‐specific antibody cocktail revealed significantly elevated expression of complex III (*P* = 0.001), complex V (*P* = 0.002), and total OXPHOS (*P* = 0.005) in liver lysates from RYGB rats compared to Sham (Fig. 3B–H). Strong trends toward increased expression were also detected for complexes I (*P* = 0.063), II (*P* = 0.055), and IV (*P* = 0.09) in RYGB rat liver lysates.

### Reactive oxygen species content and total antioxidant capacity

**Figure 4:**
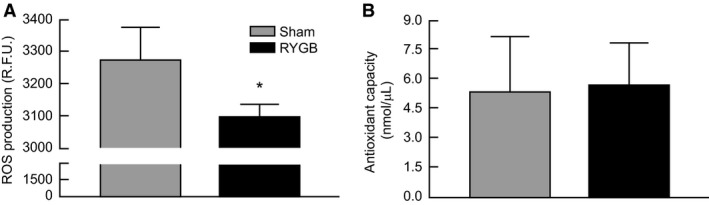
Markers of oxidative stress in the liver of Sham (N = 8) and RYGB (N = 8) animals. (A) Reactive oxygen species (ROS) production. Values represent relative fluorescence units (R.F.U.) and are normalized per 1 mg of protein. (B) Total antioxidant capacity normalized to Trolox standard. Data are expressed as mean ± STDEV. *P < 0.05 versus Sham control rats.

RYGB rats showed reduced levels of reactive oxygen species (*P* = 0.045) in liver lysates compared to Sham‐operated controls (Fig. 4A). There were no group differences in total antioxidant capacity observed between Sham and RYGB animals (Fig. 4B).

### Correlation analysis

**Figure 5:**
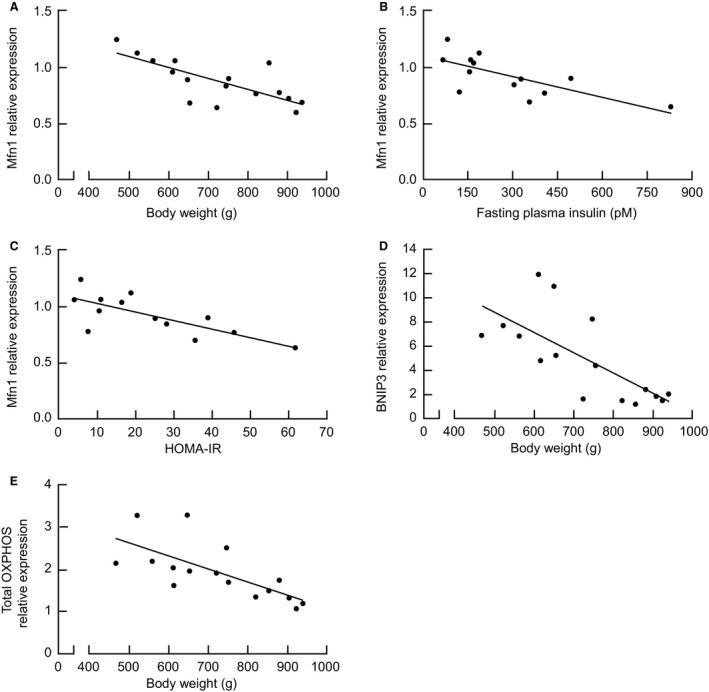
Association between Mfn1 protein expression and (A) body weight (*r* = 0.74, *P* = 0.001), (B) fasting plasma insulin (*r* = 0.71, *P* = 0.007), and (C) insulin resistance (HOMA‐IR;* r* = 0.73, *P* = 0.004) in obese Sprague‐Dawley rats. Correlation between body weight and relative protein expression of (D) BNIP3 (*r* = 0.71, *P* = 0.002) and (E) total OXPHOS complexes (*r* = 0.7, *P* = 0.003).

The metabolic and weight loss effects of diet and RYGB surgery have been published previously (Mosinski et al. 2016). Briefly, while rats in the Sham group continued to gain weight throughout the study, rats in the RYGB group lost an average of 20% of their initial body weight 90‐days postsurgery (149 ± 69 g), demonstrated a significant reduction in insulin resistance (*P* = 0.03), as measured by homeostatic model assessment (HOMA‐IR), and showed marked reductions in hepatic triglyceride accumulation (*P* < 0.0001) compared to Sham controls, despite comparable average food intake (20.6 ± 2.3 g vs. 22.9 ± 4.2 g for Sham and RYGB rats, respectively). In liver tissue (Fig. 5), we found a significant inverse correlation between Mfn1 protein expression and body weight (*r* = 0.74, *P* = 0.001), fasting plasma insulin (*r* = 0.71, *P* = 0.007), and HOMA‐IR (r = 0.73, *P* = 0.004) at 90‐days postsurgery. Specifically, concomitant with a 20% decrease in body weight and a 57% improvement in insulin sensitivity (Mosinski et al. 2016), there was a significant increase in protein expression for Mfn1 (*P* = 0.0005). Finally, reduced body weight was also associated with increased relative protein expression of BNIP3 (Fig. 5D) and total OXPHOS (Fig. 5E).

## Discussion

Bariatric surgery has emerged as one of the leading treatment options for morbid obesity and related co‐morbidities, providing effective and durable results with regard to weight loss, glycemic control and NAFLD parameters (Buchwald et al. 2004; Brethauer et al. 2011; Mingrone et al. 2015; Schauer et al. 2017). Despite significant attention, the precise mechanism that drives systemic and liver‐specific metabolic improvements following RYGB remains unknown. In this study, we investigated the effect of RYGB surgery on liver mitochondrial dynamics in SD rats fed a high‐fat diet known to induce obesity and hepatic steatosis.

Mitochondrial dysfunction has long been hypothesized to play a central role in the pathogenesis of insulin resistance and T2D (Kim et al. 2008; Rector et al. 2010; Szendroedi et al. 2011; Montgomery and Turner 2015); however, controversy still exists regarding the specific mitochondrial defect preceding the development of insulin resistance (Hoeks and Schrauwen 2012; Dela and Helge 2013). Improved hepatic mitochondrial function in response to gastric bypass has been demonstrated in recent rodent studies (Peng and Murr 2013; Verbeek et al. 2015), although data are limited. For example, RYGB resulted in increased liver expression of markers involved in mitochondrial respiration in obese SD rats (Peng and Murr 2013), and preserved respiratory chain complex I activity and restored ATP levels in high‐fat high‐sucrose fed C57BL/6 mice (Verbeek et al. 2015), yet the molecular mechanisms that exert these effects remain elusive. Normal mitochondrial quality control is mediated by events of fusion, fission, mitophagy, and biogenesis. Recently, prospective studies in mitochondrial dynamics have identified a potential mechanistic link between mitochondrial morphology and bioenergetic adaptations to metabolic status (Molina et al. 2009; Gomes et al. 2011; Distefano et al. 2017). In this study, we observed significant increases in expression of the fusion protein, Mfn1 in the liver following RYGB surgery, and elevated Mfn1 protein levels were associated with reductions in body weight and improved insulin sensitivity. Recent literature presents some conflicting results with regard to hepatic Mfn1 expression depending on the experimental model. For example, Hsu et al. (2015) reported that treating primary mouse hepatocytes with leptin resulted in increased Mfn1 expression and protected against hyperglycemia‐induced lipid accumulation, and this effect was lost with RNA silencing of Mfn1. Furthermore, leptin treatment induced liver Mfn1 expression and normalized serum lipid profiles in mice fed a high‐fructose diet (Hsu et al. 2015). These findings are in agreement with this study, indicating that enhanced Mfn1 expression in the liver provides beneficial metabolic effects. In contrast, Kulkarni et al. (2016) found that liver‐specific ablation of Mfn1 in mice was protective against high‐fat diet‐induced insulin resistance, despite an increase in mitochondrial fragmentation, ROS production, and lipid droplet size. The observed increase in mitochondrial fusion proteins in this study, in the absence of any changes in markers of fission, indicate that mitochondria may adapt to an altered metabolic environment by a finely tuned molecular control mechanism in response to RYGB surgery, even in the face of high‐fat feeding.

BNIP3 is a member of the Bcl‐2 protein family of cell death regulators, and a non‐canonical promoter of mitophagy (Landes et al. 2010; Zhang et al. 2016). Interestingly, we observe a significant induction of liver BNIP3 expression following RYGB surgery, which was initially surprising given the lack of change in Parkin and PINK1 expression, and noted increases in markers of mitochondrial biogenesis (PGC1*α*, NRF1) and respiratory complex content. Furthermore, although BNIP3 is generally proposed to activate autophagy and cell death pathways (Tracy et al. 2007), we have previously reported reduced apoptosis in the liver of the same obese rats following RYGB surgery compared to Sham animals (Mosinski et al. 2016). These results suggest that BNIP3 may play a role in mitochondrial quality control independent of mitophagy, and future studies will be necessary to delineate whether alternative signaling pathways are involved. Glick et al. (2012) have previously shown that BNIP3 is highly expressed in rodent liver, and BNIP3 knockout mice develop excess hepatic lipid and ROS accumulation, as well as steatohepatitis. These findings are in agreement with the current study in that BNIP3 may be essential in mediating the reduced liver triglyceride content and oxidative stress in response to gastric bypass surgery.

NAFLD is a common co‐morbidity in patients with obesity or T2D, whereby approximately 70% of obese patients with T2D are reported to also have NAFLD (Portillo‐Sanchez et al. 2015). The liver is a central hub of metabolic activity in the body, and in the setting of obesity and hepatic insulin resistance, triglyceride accumulation occurs in response to the altered nutrient environment (Rector et al. 2008; Cusi 2012). Chronic exposure to excess nutrient supply is known to initiate ER stress in multiple tissues, including the liver (Flamment et al. 2012; Bravo et al. 2013). We have previously shown that RYGB normalizes hepatic steatosis and ER stress in SD rats maintained on a high‐fat diet for 90‐days postoperatively (Mosinski et al. 2016), and several recent reports have determined that ER stress and altered mitochondrial dynamics are intimately linked (de Brito and Scorrano 2008; Grimm 2012; Marchi et al. 2014). For example, Sebastian et al. (2012) demonstrated that liver‐specific Mfn2 knockout mice manifest with hepatic and peripheral insulin resistance, elevated H_2_O_2_ levels, and increased markers of ER stress. In vivo studies in both humans and rodent models with NAFLD have demonstrated that the progression from simple steatosis to NASH occurs in response to a dysfunction in mitochondrial oxidative flux (Koliaki et al. 2015; Cusi 2016; Patterson et al. 2016). Upregulated and incomplete mitochondrial oxidation leads to the production of ROS and inflammation, as well as an accumulation of toxic lipid intermediates (Rector et al. 2008; Sunny et al. 2017). Inhibition of mitochondrial fusion is associated with impaired oxidative phosphorylation and increased ROS generation (Liesa and Shirihai 2013; Wai and Langer 2016). The absence of differences in total antioxidant capacity between Sham and RYGB liver extracts in the present study suggests that reduced hepatic ROS content in the RYGB group is more likely due to decreased production, rather than enhanced scavenging of free radicals and toxic species. Our observed decrease in hepatic ROS production in RYGB animals compared to Sham controls further supports evidence suggesting that reduced oxidative stress may be a key contributor for ameliorating insulin resistance (Bonnard et al. 2008).

Metabolic homeostasis is regulated by cellular adaptations to nutrient status, often via transcriptional events. Here, we observed increased expression of the transcriptional regulator of mitochondrial biogenesis, PGC1*α* both at the mRNA and protein level in the livers of RYGB animals compared to Sham controls. These findings align with a previous report by Gastaldi et al. (2007) who observed increased PGC1*α* expression in skeletal muscle following RYGB surgery in 17 obese females. Interestingly, both Mfn1 and Mfn2 are proposed to be downstream targets of PGC1*α* (Cartoni et al. 2005; Hsu et al. 2015). While changes in liver Mfn2 protein levels did not reach statistical significance in the present study, the strong trend toward increased Mfn2 protein expression coupled with significant increases in Mfn1 following RYGB suggest PGC1*α* may exert its effects on the liver via mitochondrial fusion.

In conclusion, this work identifies a new mechanism by which gastric bypass may confer global improvements in insulin sensitivity and reduce hepatic triglyceride accumulation. To our knowledge, this is the first investigation of RYGB‐induced alterations in liver mitochondrial dynamics in the presence of an obesogenic diet. We demonstrate that RYGB increases the expression of mitochondrial fusion protein, Mfn1 and mitophagy marker, BNIP3 in the liver of SD rats exposed to a chronic high‐fat diet, and elevated Mfn1 levels are associated with reductions in body weight and improved insulin sensitivity. Furthermore, the observed increases in hepatic PGC1*α* and NRF1 expression, citrate synthase activity and mitochondrial respiration complex content in the SD animal model align with previous findings from rodent and human studies evaluating the effects of RYGB on mitochondrial biogenesis in skeletal muscle (Gastaldi et al. 2007) and adipose tissue (Jahansouz et al. 2015). More prospective research utilizing direct and functional methods (such as confocal and electron microscopy) to assess mitochondrial morphology and dynamics in response to RYGB will be beneficial in strengthening the validity of our findings. Future studies targeting Mfn1 in the setting of weight loss surgery will better define the mechanisms that link mitochondrial architecture to bioenergetic adaptations in liver, and mitochondrial dynamics proteins may emerge as a new functional target for the treatment of obesity and liver‐related disorders.

## Conflict of Interest

The authors report no conflicts of interest relative to this work.
